# Full Genome Sequence of Egg Drop Syndrome Virus Strain FJ12025 Isolated from Muscovy Duckling

**DOI:** 10.1128/genomeA.00623-13

**Published:** 2013-08-22

**Authors:** Guanghua Fu, Hongmei Chen, Yu Huang, Longfei Cheng, Qiuling Fu, Shaohua Shi, Chunhe Wan, Cuiteng Chen, Jiansheng Lin

**Affiliations:** Institute of Animal Husbandry and Veterinary Medicine, Fujian Academy of Agricultural Sciences, Fuzhou, Fujian, Chinaa; Fujian Animal Diseases Control Technology Development Center, Fuzhou, Fujian, Chinab

## Abstract

Egg drop syndrome virus (EDSV) strain FJ12025 was isolated from a 9-day-old Muscovy duckling. The results of the sequence showed that the genome of strain FJ12025 is 33,213 bp in length, with a G+C content of 43.03%. When comparing the genome sequence of strain FJ12025 to that of laying duck original strain AV-127, we found 50 single-nucleotide polymorphisms (SNPs) between the two viral genome sequences. A genomic sequence comparison of FJ12025 and AV-127 will help to understand the phenotypic differences between the two viruses.

## GENOME ANNOUNCEMENT

Egg drop syndrome virus (EDSV) was first reported in chickens in the 1970s (1). This virus is a member of the genus *Atadenovirus* under the family *Adenoviridae*, and it has also been known as duck adenovirus 1 (DAdV-1), duck adenovirus A, adenovirus 127, and egg-drop-syndrome-76 (EDS-76) virus (2). Although it has almost been eliminated from commercial breeders, sporadic outbreaks of egg drop syndrome are still reported. EDSV mainly causes sudden drop in egg production in laying birds accompanied by a reduction in egg quality. In 2001, a case was reported in young goslings with severe acute respiratory disease in Hungary, and the infectious agent was confirmed to be the EDSV by the viral isolation, serological and genomic examinations, and experimental infection of 1-day-old goslings (3). Cha et al. (4) also found that EDSV caused severe acute respiratory symptoms in 10-day-old Pekin ducklings.

To date, there is only one full-genome sequence of EDSV deposited in the GenBank database, and this genomic information is derived from EDSV strain AV-127 (accession no. Y09598), which was isolated from laying birds (5). We report here the full-genome sequence of EDSV strain FJ12025, which was identified from a 9-day-old Muscovy duckling and is characterized by congestion with varying degrees of hyperemia in the liver. Viral DNA was extracted from the allantoic fluid of embryonated duck eggs infected with FJ12025 by using a QIAamp DNA blood mini kit (Qiagen, Valencia, CA) according to the manufacturer’s instructions. Overlapping fragments of the viral genome sequence were generated by PCR using 22 pairs of primers. Overlapping amplicons were purified and cloned into pMD18-T vector (TaKaRa) and sequenced with genomic analyzer 3730xl (Applied Biosystems). Sequences of the cloned fragments were assembled using the SeqMan programs (Lasergene 7.1; DNAStar, Madison, WI). Gene annotation of the EDSV genome was transferred from the strain AV-127 genome (accession no. Y09598) using the Genome Annotation Transfer Utility (GATU) software (6).

The genome of strain FJ12025 is 33,213 bp in length, with a G+C content of 43.03%. It possesses a genomic organization similar to that of EDSV strain AV-127. Twenty-nine genes were annotated, which identified a high homology to other known adenovirus proteins. Phyologenetic analysis based on the main proteins indicated that FJ12025 has a close genetic relationship to ovine adenovirus D, bovine adenovirus D, and snake adenovirus, but is distinct from the aviadenovirus, which has also been confirmed by Hess et al. (5). Compared to the conference strain AV-127, 49 single-nucleotide polymorphisms (SNPs) were found in the genome sequence. The genome sequence comparison of strain FJ12025 and AV-127 will help to understand the phenotypic differences between these two viruses.

### Nucleotide sequence accession number. 

The complete genome sequence of egg drop syndrome virus strain FJ12025 was deposited in GenBank under accession no. KF286430.

## References

[1] Van Eck JH Davelaar FG Heuvel-Plesman TA Van Kol N Kouwenhoven B Guldie FH 1976 Dropped egg production, soft shelled and shell-less eggs associated with appearance of precipitins to adenovirus in flocks of laying fowls. Avian Pathol. 5:261–272 18777355 10.1080/03079457608418195

[2] Harrach B Benkö M Both GW Brown M Davison AJ Echavarría M Hess M Jones MS Kajon A Lehmkuhl HD Mautner V Mittal SK Wadell G 2012 Family *Adenoviridae*, p 125–141 *In * King AMQ Adams MJ Carstens EB Lefkowitz EJ (ed), Virus taxonomy. Ninth report of the International Committee on Taxonomy of Viruses. Elsevier Academic Press, Amsterdam, The Netherlands

[3] Ivanics E Palya V Glavits R Dan A Palfi V Revesz T Benko M 2001 The role of egg drop syndrome virus in acute respiratory disease of goslings. Avian Pathol. 30:201–208 19184901 10.1080/03079450120054604

[4] Cha SY Kang M Moon OK Park CK Jang HK 2013 Respiratory disease due to current egg drop syndrome virus in Pekin ducks. Vet. Microbiol. 165:305–311 23639475 10.1016/j.vetmic.2013.04.004

[5] Hess M Blöcker H Brandt P 1997 The complete nucleotide sequence of the egg drop syndrome virus: an intermediate between mastadenoviruses and aviadenoviruses. Virology 238:145–156 9375018 10.1006/viro.1997.8815

[6] Tcherepanov V Ehlers A Upton C 2006 Genome annotation transfer utility (GATU): rapid annotation of viral genomes using a closely related reference genome. BMC Genomics 7:150. 10.1186/1471-2164-7-150 16772042 PMC1534038

